# Patterns and predictors of orbitofrontal sulcogyral morphology in a nonclinical population

**DOI:** 10.1162/imag_a_00389

**Published:** 2024-12-19

**Authors:** Marisa A. Patti, Donielle Beiler, Will Snyder, Shane Kozick, Vanessa Troiani

**Affiliations:** AJ Drexel Autism Institute, Philadelphia, PA, United States; Geisinger Autism and Developmental Medicine Institute, Lewisburg, PA, United States

**Keywords:** orbitofrontal cortex, OFC, sulcogyral patterns

## Abstract

Less common orbitofrontal cortex (OFC) sucogyral patterns are observed at higher rates among those witth psychopathology. Previous work has assumed demographic characteristics have no influence on OFC sulcogyral patterns. However, the influence of sociodemographic and health-related characteristics on OFC patterns within a neurotypical population has not been formally evaluated. We used structural brain magnetic resonance imaging (MRI) from a cohort from the Human Connectome Project (HCP) with existing OFC sulcogyral characterizations (n = 238); none of the participants had psychiatric diagnoses. We evaluated distributions of participant demographics (i.e., age), socioeconomic factors (i.e., employment), and health history-related factors (i.e., smoking history) by OFC sulcogyral pattern within each hemisphere. We then used logistic regression to estimate the odds of OFC sulcogyral pattern by participant characteristics. Distributions of study sample characteristics did not vary substantially by OFC sulcogyral pattern type within either hemisphere. Findings from logistic regression analyses suggest no association between OFC sulcogyral pattern and any of the demographic or socioeconomic characteristics. Two health history-related characteristics, body mass index (BMI) and smoking history, were associated with increased odds of having specific OFC pattern types. For example, individuals with obesity had 2.65 increased odds (95% CI: 1.17, 6.65) of having OFC sulcogyral pattern Type II, III, or IV, compared with Type I in the left hemisphere with normal BMIs. We did not observe substantial influence of demographic or socioeconomic characteristics on OFC sulcogyral patterns. These results confirm assumptions made in previous work that demographic and socioeconomic characteristics do not seem to impact OFC patterns. We do show some evidence for an influence of health history-related factors (obesity and smoking history); future work should evaluate whether these and other phenotypic risk factors interact to modify the relationship between psychiatric diagnoses and OFC sulcogyral patterns.

## 1 Introduction

The orbitofrontal cortex (OFC) is a brain region associated with a range of higher-order behaviors, including multimodal integration, emotional processing, motivation, decision making, and goal-directed behavior (Gottfried et al., 2003;Holland & Gallagher, 2004;Kringelbach, 2005;Ongür & Price, 2000;Walton et al., 2004). Differences in brain morphology, such as surface morphology, within the OFC have been linked to individual differences in behavior (Nakamura et al., 2019;Rudebeck & Rich, 2018;Zhang et al., 2016). The surface of the brain consists of folded configurations, formed by sulci and gyri (grooves and ridges), that create an individually unique sulcogyral landscape. The development of sulcogyral morphology is thought to begin*in utero*based on evidence that primary, secondary, and tertiary cortical folds emerge at 20, 32, and 34 weeks gestation, respectively (Cachia et al., 2021;Chi et al., 1977;Dubois et al., 2008,^2019^;Habas et al., 2012;Rajagopalan et al., 2011), and the sulcogyral patterns in the OFC as well as other brain regions are believed to be consistent through the life course (Armstrong et al., 1995;Cachia et al., 2016), though this has not been explicitly studied. Despite extensive individual variation in sulcogyral morphology across the brain, within the OFC, a group of sulci, referred to as the “H-sulcus” can be classified consistently into one of four patterns. Originally codified by Chiavares and Petrides (2000) and later expanded upon, four OFC pattern types have been identified based on the continuity and discontinuity of the medial orbital sulcus (MOS) and lateral orbital sulcus (LOS) (Chakirova et al., 2010). Pattern types are named according to their frequency within the general population, with Type I being the most common (~60%), relative to Type II (~25%), and Type III (~10%), followed by Type IV (~5%).

Prior work has identified elevated frequencies of less common pattern types (i.e., Type III) among those with schizophrenia, (Chakirova et al., 2010;Isomura et al., 2017;Lavoie et al., 2014;Nakamura et al., 2007;Takayanagi et al., 2010) and subsequently other psychiatric conditions such as bipolar disorder, autism spectrum disorder, and gambling disorder (Li et al., 2019;Patti & Troiani, 2018;Watanabe et al., 2014). Interestingly, altered distributions in the frequencies of OFC patterns are not observed in all psychiatric diagnoses, as null associations were observed among those with obsessive compulsive disorder, cannabis use disorder, and cocaine use disorder (Chye et al., 2017;Delahoy et al., 2019;Patti et al., 2020). Associations between OFC pattern types and psychopathology are derived based on comparisons of pattern type frequencies observed among groups of cases and controls. Indeed, an individual with diagnosed schizophrenia may still have a common OFC pattern (i.e., Type I) in both hemispheres of the brain. Thus, it is currently suggested that less common OFC pattern types represent risk for transdiagnostic traits associated with psychopathology, rather than serving as a biomarker indicating a specific psychiatric diagnosis. However, it is still unclear whether the manifestation of psychiatric symptoms is attributed to the presence of an uncommon pattern type (i.e., Type III) or the lack of a protective pattern type (i.e., Type I) (Bartholomeusz et al., 2013;Chakirova et al., 2010;Lavoie et al., 2014). Some work has also observed that the presence of an uncommon pattern type in the right hemisphere may be more strongly associated with psychopathology compared with the left hemisphere, though these findings are not consistent across studies (Chakirova et al., 2010;Lavoie et al., 2014;Patti & Troiani, 2018;Yoshimi et al., 2016). Much less is known regarding the association between OFC pattern types and distributions of demographic characteristics (i.e., age or sex), socioeconomic factors (i.e., income and employment status), or health history-related factors (i.e., smoking status or BMI). For example, prior work has speculated that those with less common pattern types are more likely to have lower socioeconomic status and have lower cognitive abilities; however, this has not been consistently reported, or evaluated in control populations (Lavoie et al., 2014;Nakamura et al., 2007). It may be possible that this proposed association is indirectly attributed to modification by specific characteristics, such that those with more severe psychopathology may be less likely to hold consistent employment (Eaton et al., 2008;Patti & Troiani, 2018). Some studies that observed differences between their case and control samples, for example, older patients with schizophrenia who smoke, dismissed the potential impact of smoking on OFC pattern type, given that OFC patterns are suspected to be codified early in life (Uehara-Aoyama et al., 2011). In other cases, we have no reason to believe that frequency of OFC patterns would vary by certain characteristics, such as sex (Uehara-Aoyama et al., 2011) or race. In fact, our group recently observed no difference in OFC pattern distribution among control subjects in an entirely Black sample, relative to distributions previously reported among predominantly White control samples (Patti et al., 2020). Yet, because all of the previous work beyond the seminal paper has been completed in case–control designs, the relationship between various demographic, sociodemographic, and health-related characteristics, and OFC sulcogyral pattern type has not yet been statistically evaluated within a nonclinical sample.

Here, we aim to evaluate the patterns and predictors of study participant demographic, socioeconomic, and health history-related characteristics by OFC sulcogyral pattern types, using a large, diverse sample of individuals with previously established pattern types from the Human Connectome Project (HCP). While we do not expect there to be variations in OFC sulcogyral pattern types across some demographic characteristics, such as sex and race, we are interested in exploring whether differences in OFC pattern type exist across a range of population characteristics. Thus, our primary goal is to describe these patterns within a control sample of participants with no diagnosed psychopathology, rather than explicit hypothesis testing.

## 2 Methods

### 2.1 Study sample participants

We obtained high-resolution structural brain images from the Human Connectome Project (HCP), a publicly available data set. All participants provided their written informed consent for their involvement in the HCP. Of note, participants were excluded from the original study if they were diagnosed with psychiatric, neurological, or cardiovascular disease, or had a history of substance abuse, and/or hospitalization (additional details available inVan Essen et al., 2012). We restricted our sample to only include those participants within twin pairs among this data set (n = 476 individuals), who had available OFC sulcogyral tracing and characterization data completed as part of previous work. We randomly selected only one twin per pair to be included in these analyses (n = 238), with OFC pattern identification methods previously described (Troiani et al., 2022). To increase the number of participants included with uncommon pattern types (Types II–IV), in cases where one twin had bilateral Type I, we selected the other twin to be included in analyses. This is reflected in the difference in the distribution of pattern types between the full sample versus the restricted sample.

### 2.2 Image acquisition, preprocessing, and sulcal pattern pipeline

Methods for image acquisition, preprocessing, and sulcal pattern classification for this sample have been previously defined (Troiani et al., 2022). Briefly, we leveraged existing data obtained from the HCP S1200 release (Van Essen et al., 2012) using outputs from both the*PreFreeSurfer*and*FreeSurfer*Pipelines (Glasser et al., 2013). All participants were scanned using a 3T “Connectome Skyra” scanner at Washington University, St. Louis (Gao et al., 2021). From the*PreFreeSurfer*pipeline, we utilized the T1 weighted MPRAGE aligned to standard MNI space (TR 2400 ms, TE 2.14 ms, flip angle = 8°, FOV 224 × 224 mm^2^, voxel size = 0.7 mm isotropic, scan time = 7:40 min) to retrieve an image of the cortical gray matter ribbon (converted into NIFTI format). OFC sulcal extraction using FreeSurfer inputs was performed by BrainVISA software (Riviere et al., 2003) using a previously validated pipeline for extracting binary images of OFC sulcal groups (Snyder et al., 2019).

### 2.3 Categorical analysis of sulcal patterns

**Fig. 1.**
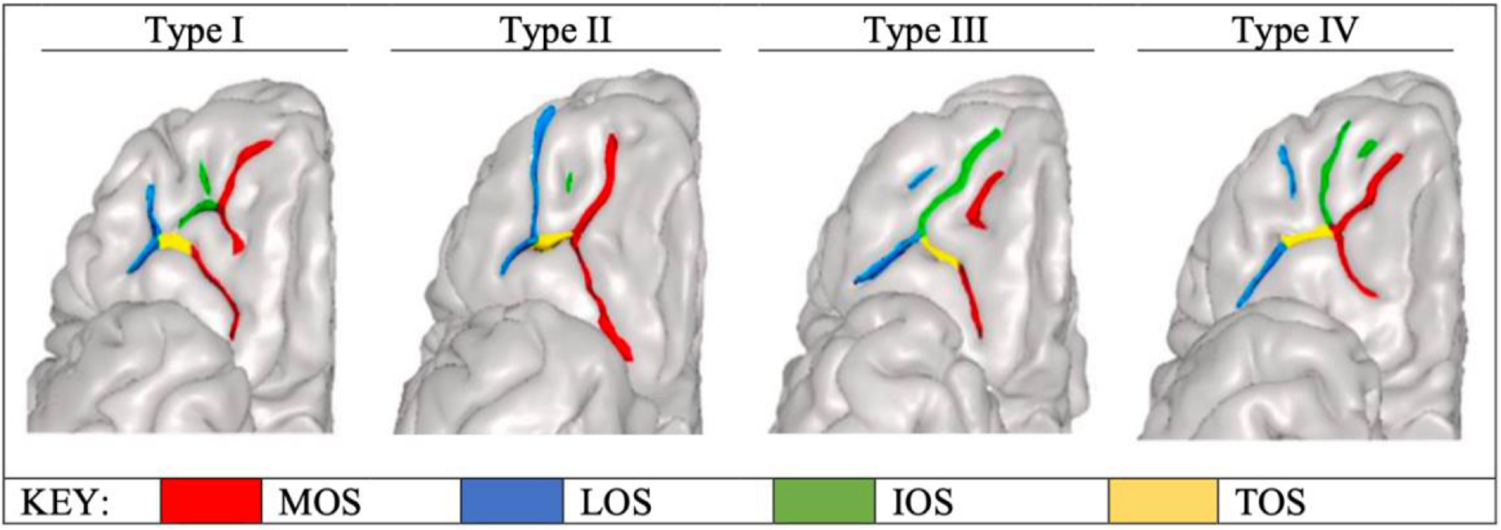
Four orbitofrontal cortex pattern types. MOS: medial orbital sulcus; LOS: lateral orbital sulcus; IOS: intermediate orbital sulcus; TOS: transverse orbital sulcus. Four examples each of Types I, II, III, and IV patterns, with individual orbitofrontal sulci labeled. The Type I pattern has a discontinuous MOS and continuous LOS, Type II a continuous MOS and continuous LOS, Type III a discontinuous MOS and discontinuous LOS, and Type IV a continuous MOS and discontinuous LOS. Right hemisphere only is presented in the figure. This figure has been adapted from our prior work (Troiani et al., 2022).

Consistent with prior work (Patti et al., 2020;Patti & Troiani, 2018;Snyder et al., 2019;Troiani et al., 2022), OFC sulcogyral pattern type was based on the continuity or discontinuity of the medial orbital sulcus (MOS) and lateral orbital sulcus (LOS) within each hemisphere of the brain. For example, characterization of Type I consists of a continuous LOS and discontinuous MOS, Type II having both a continuous MOS and LOS, Type III having both a discontinuous MOS and LOS, and Type IV having a continuous MOS and discontinuous LOS (Fig. 1).

As previously described (Troiani et al., 2022), two raters (M.A.P. and W.S.) independently characterized pattern type using established procedures. In cases of disagreement, raters reviewed the subject together until a consensus was reached. Of note, true disagreement among raters was rare; rather, disagreement more often stemmed from human errors such as misidentification of a sulcus and/or failure to apply appropriate rules for individual sulcus determination. To calculate inter-rater reliability consistent with other published work that only uses 1 primary rater, we randomly selected 20 hemispheres and a third rater (V.T.) characterized overall patterns. This inter-rater reliability was high (kappa: 0.85).

### 2.4. Participant characteristics

We selected multiple characteristics available from the HCP. These include demographic characteristics such as age, gender, race and ethnicity, and socioeconomic factors including employment status, annual total household income, and total years of education. We also considered several health history-related factors such as body mass index (BMI, based on self-reported values of height and weight), smoking history, and parent history of psychiatric disorders assessed via self-report on standardized questionnaires. We determined handedness based on continuous scores (range -100 to 100) on the Edinburgh Handedness Questionnaire (Oldfield, 1971), where positive scores indicate a subject is more right handed, and negative scores indicate a subject is more left handed. We used the threshold of >70 to determine whether a subject was right handed, where scores≤70 were considered to be not right handed (Schachter et al., 1987). Cognition was evaluated using the Cognitive Function Composite Score National Institute of Health (NIH) toolbox, where higher scores indicate higher levels of cognitive functioning (Akshoomoff et al., 2013).

### 2.5 Statistical analyses

We first conducted exploratory analyses to examine the distributions of OFC sulcogyral pattern types within the entire sample, and by hemisphere. Given that less common pattern types (Types II, III, and IV) are all previously associated with psychopathology, and due to sample size limitations, we collapsed categories to two groups: common type (Type I) and uncommon types (Types II, III, and IV). Next, we evaluated distributions of study sample demographic, socioeconomic, and health history-related characteristics by OFC sulcogyral pattern types and among the common versus uncommon subsets. Using logistic regression, we calculated the odds of OFC pattern type category based on each of these characteristics, examining each variable in a separate model for the right and left hemispheres separately. The common type group (Type I) served as the reference group. We also applied Bonfferoni corrections to adjust for multiple comparisons.

Since the examination of these patterns in the context of human health and disease, the pattern types have been distinguished based on specific type, with Type I thought to be protective (Chakirova et al., 2010). Following the seminal paper on the original three OFC pattern types, Type IV was identified (Chakirova et al., 2010). Due to their rarity as well as sharing common features of a discontinuous LOS, Type III and Type IV are often grouped together in statistical analyses. While the OFC pattern types are well established, there are still relatively few studies examining them in large populations, and much knowledge remains to be learned regarding their role in behavior and psychiatric illness. For example, specific underlying features, such as sulcus discontinuity, may map onto brain–behavior relationships better than the overall pattern types in that they could be based on more global patterns formed from several intersecting sulci. Thus, as our primary analysis and research questions were exploratory in nature, we conducted several secondary analyses.

Individual H-shaped OFC sulcogyral patterns are differentiated by the continuity of the MOS and LOS; however, it remains unclear whether the continuity of both sulci is meaningful in their association with psychopathology, or whether assessing the continuity of only one sulcus would be sufficient. For this reason, we conducted all primary analyses by comparing those with a continuous LOS (Types I and II) with those with a discontinuous LOS (Type III and Type IV). While Type I and Type II have not been previously categorized together, Types III and IV are often combined, based on the similar characteristic of a discontinuous LOS, rare prevalence, and associations with psychopathology (Patti et al., 2020). The combined category of those with a continuous LOS (Type I and Type II) served as the reference group.

We used R version (4.1.0) for all statistical analyses (R Core Team, 2015).

## 3 Results

### 3.1 Distributions of OFC pattern types

**Fig. 2.**
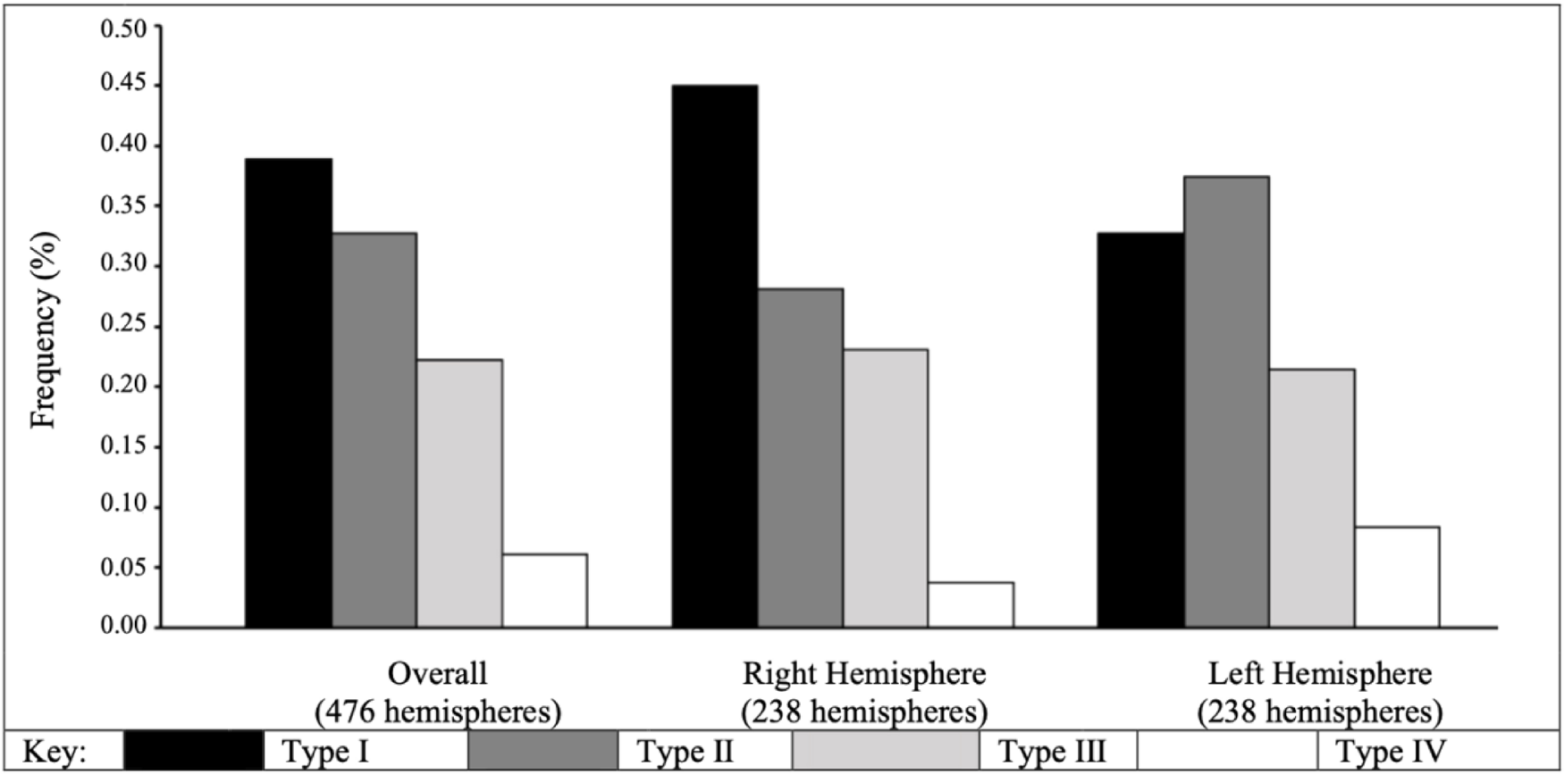
Hemispheric OFC pattern type frequency. OFC: orbitofrontal sulcus. Type I pattern has a discontinuous MOS and continuous LOS, Type II a continuous MOS and continuous LOS, Type III a discontinuous MOS and discontinuous LOS, and Type IV a continuous MOS and discontinuous LOS. Note, our sample size includes n = 238 participants for a total of 476 hemispheres.

Even in our subselected sample that prioritized non-Type I twins, we observed Type I to be the most common pattern type (overall frequency: 39%), followed by Type II (33%), Type III (22%), and Type IV (6%) (Fig. 2,Supplemental Table S1). This pattern of distribution is consistent in the right hemisphere, however, we observe more variation in the distribution of OFC pattern type in the left hemisphere, such that Type II occurs more frequently than Type I. However, this distribution of OFC pattern type can be attributed to the selection of participants (one participant from each twin pair). Of note, within the full sample (including both twin pairs), Type I was the most common pattern type overall, and within each hemisphere (for information on OFC pattern type distributions within the original full cohort, seeSupplemental Table S1).

**Fig. 3.**
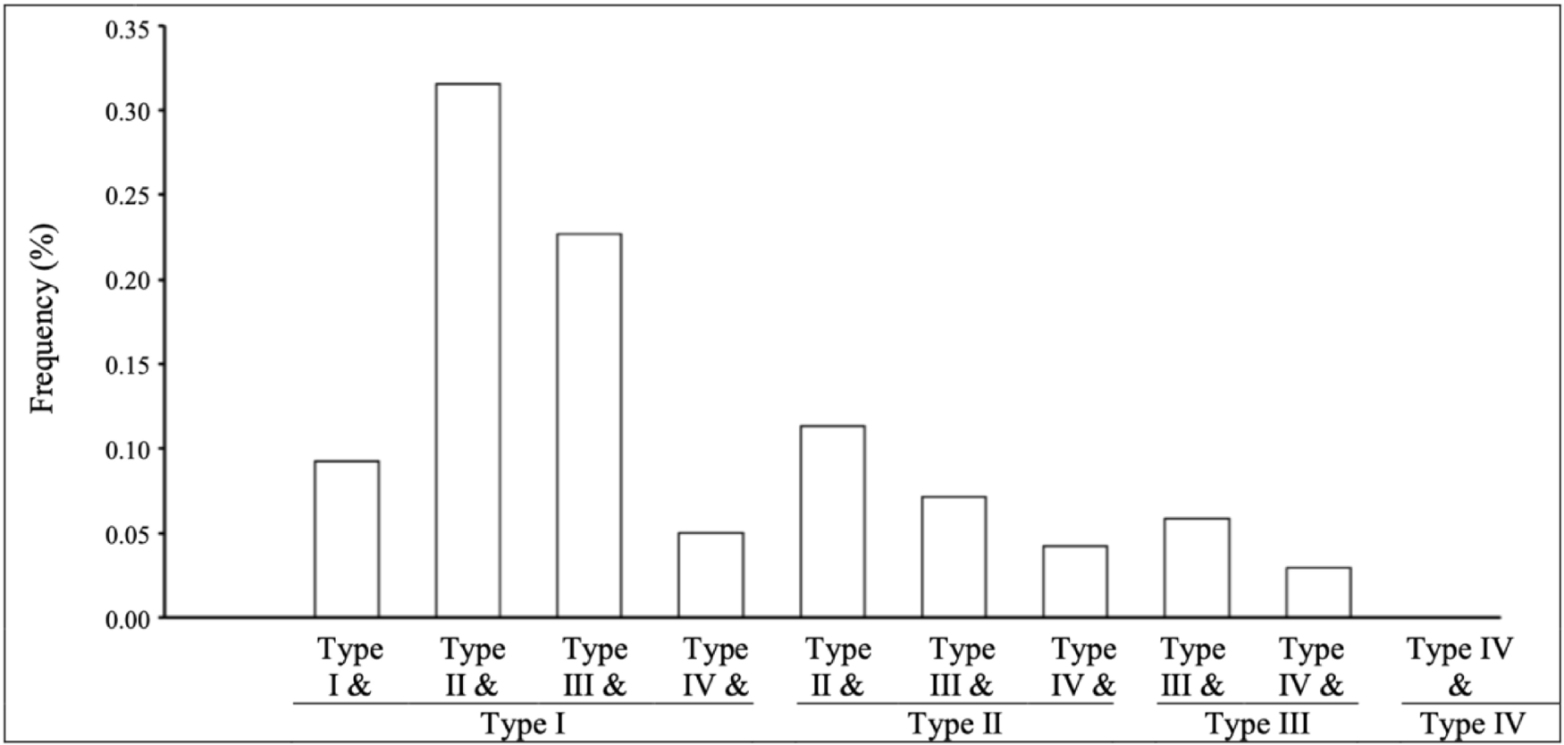
Joint hemispheric OFC pattern type frequency. OFC: orbitofrontal sulcus. Type I pattern has a discontinuous MOS and continuous LOS, Type II a continuous MOS and continuous LOS, Type III a discontinuous MOS and discontinuous LOS, and Type IV a continuous MOS and discontinuous LOS. Note, our sample size includes n = 238 participants.

When considering both hemispheres together, it was most common for a participant to have at least one Type I pattern type in either hemisphere paired with a less common pattern type (Type II, Type III, or Type IV, referred to hereafter as “uncommon”) (Fig. 3). While participants rarely had two of the same pattern types in both hemispheres, it was more likely to observe bilateral Type II or Type III among those who did not have a Type I pattern in either hemisphere. For example, it was more common for a participant to have bilateral Type II (11%) than Type II with Type III (4.2%) or Type II with Type IV (5.9%) (Supplemental Table S1).

### 3.2 Distributions of study sample characteristics

Overall, participants were primarily female, non-Hispanic White, between the ages of 25 and 35 years, were employed full-time, with annual incomes >$75 k (Table 1). The majority of participants had 13–16 years of education, were right handed, with normal or underweight BMIs, and never smoked. While few participants reported parental history of bipolar disorder, roughly 30% reported any parent had at least one psychiatric diagnosis (including schizophrenia or psychosis, depression, bipolar, anxiety, drug or alcohol problems, Alzheimer’s or dementia, Parkinson’s, or Tourette’s syndrome). Distributions of study sample characteristics did not vary between those with a Type I OFC sulcogyral pattern on the right or left hemisphere compared with those with Type II, Type III, or Type IV, except for BMI or smoking history. For example, the highest frequency of regular smokers in the right hemisphere was among those participants with Type I, but in the left hemisphere, those with a Type II, Type III, or Type IV had the highest frequency of regular smokers. In the left hemisphere, there was a higher frequency of participants with Type II, Type III, or Type IV, compared with Type I, who had a BMI in the obese range. We observed similar patterns in the distribution of study sample characteristics by pattern type (Type I v. Type II v. Type III and Type IV) (Supplemental Table S2). When comparing distributions of study sample characteristics between the twins included in the analytic sample compared with those who were excluded, we did not observe any substantial differences (Supplemental Table S3).

**Table 1.** Description of participant characteristics by hemisphere pattern type.

		Right hemisphere		Left hemisphere	
	Full sample	Type I	Other	Type I	Other
Variable	N (%)	N (%)	N (%)	N (%)	N (%)
	238 (100)	107	131	78	160
Age					
<25	21 (8.9)	9 (8.4)	12 (9.2)	7 (9.0)	14 (8.9)
25–30	100 (42)	46 (43)	54 (42)	32 (41)	86 (54)
30–35	100 (42)	45 (42)	55 (42)	35 (45)	65 (41)
>35	17 (7.1)	7 (6.5)	10 (7.6)	4 (5.1)	13 (8.1)
Gender					
Female	145 (61)	66 (62)	79 (60)	51 (65)	94 (59)
Male	93 (39)	41 (38)	52 (40)	27 (35)	66 (41)
Race					
White	197 (83)	88 (82)	109 (83)	66 (85)	131 (82)
Black	26 (11)	13 (12)	13 (9.9)	6 (7.7)	20 (13)
Other ^a^	15 (6.3)	6 (5.6)	9 (6.9)	6 (7.7)	9 (5.6)
Ethnicity					
Hispanic or Latino	6 (2.5)	3 (2.8)	3 (2.2)	3 (3.8)	3 (1.9)
Not Hispanic or Latino	229 (96)	103 (96)	126 (96)	74 (95)	155 (97)
Unknown or not reported	3 (1.3)	1 (0.9)	2 (1.5)	1 (1.3)	2 (1.3)
Employment					
Full-time	162 (68)	75 (70)	87 (66)	49 (63)	113 (71)
Part-time	37 (16)	13 (12)	24 (18)	15 (19)	22 (14)
Not working	39 (16)	19 (18)	10 (15)	14 (18)	25 (16)
Household Income					
<$30 k	55 (23)	28 (26)	27 (21)	18 (23)	37 (23)
$30 k–<$75 k	73 (31)	29 (27)	44 (34)	19 (24)	54 (34)
>$75 k	110 (46)	50 (47)	60 (46)	41 (53)	69 (43)
Education ^b^					
<12 years	40 (17)	19 (18)	21 (16)	12 (15)	28 (18)
13–16 years	153 (64)	70 (65)	83 (63)	52 (67)	101 (63)
17+ years	45 (19)	18 (17)	27 (21)	14 (18)	31 (19)
Handedness					
Right handed	156 (66)	65 (61)	91 (69)	56 (72)	100 (63)
Not right handed	82 (34)	42 (39)	40 (31)	22 (28)	60 (38)
BMI					
Normal or underweight ^c^	105 (44)	47 (44)	58 (44)	37 (47)	68 (43)
Overweight	86 (36)	40 (37)	46 (35)	33 (42)	53 (33)
Obese	47 (20)	20 (19)	27 (21)	8 (10)	39 (24)
Smoking history ^d^					
Never smoked	130 (55)	54 (50)	76 (58)	43 (55)	87 (54)
Previous or some smoking	43 (18)	15 (14)	28 (21)	19 (24)	24 (15)
Regular smoking	65 (27)	38 (36)	27 (21)	16 (21)	49 (31)
Parental psychiatric history ^e^					
History of bipolar disorder ^f^	8 (3.4)	3 (2.8)	5 (3.8)	2 (2.6)	6 (3.8)
No parental psychiatric history ^g^	164 (69)	68 (64)	96 (73)	57 (73)	107 (67)
Any parental psychiatric history	74 (31)	39 (36)	35 (27)	21 (27)	53 (33)

OFC: orbitofrontal cortex; BMI: body mass index.

a Includes “more than one,” “unknown/unreported,” and “Asian/native Hawaiian/other pacific islander”.

b Consistent with typical time for: less than high school or high school graduate, some college or trade school, advanced degree.

c Only n = 6 participants were underweight.

d Previous or smoke smoking (1–99 times, experimental smoking).

e Mother or father psychiatric history.

f Mother history (n = 5), father history (n = 3).

g Psychiatric history includes one of the following: schizophrenia or psychosis, depression, bipolar, anxiety, drug or alcohol problems, Alzheimer’s or dementia, Parkinson’s, Tourette’s syndrome.

### 3.3 Odds of OFC pattern type based on study sample characteristics

**Fig. 4.**
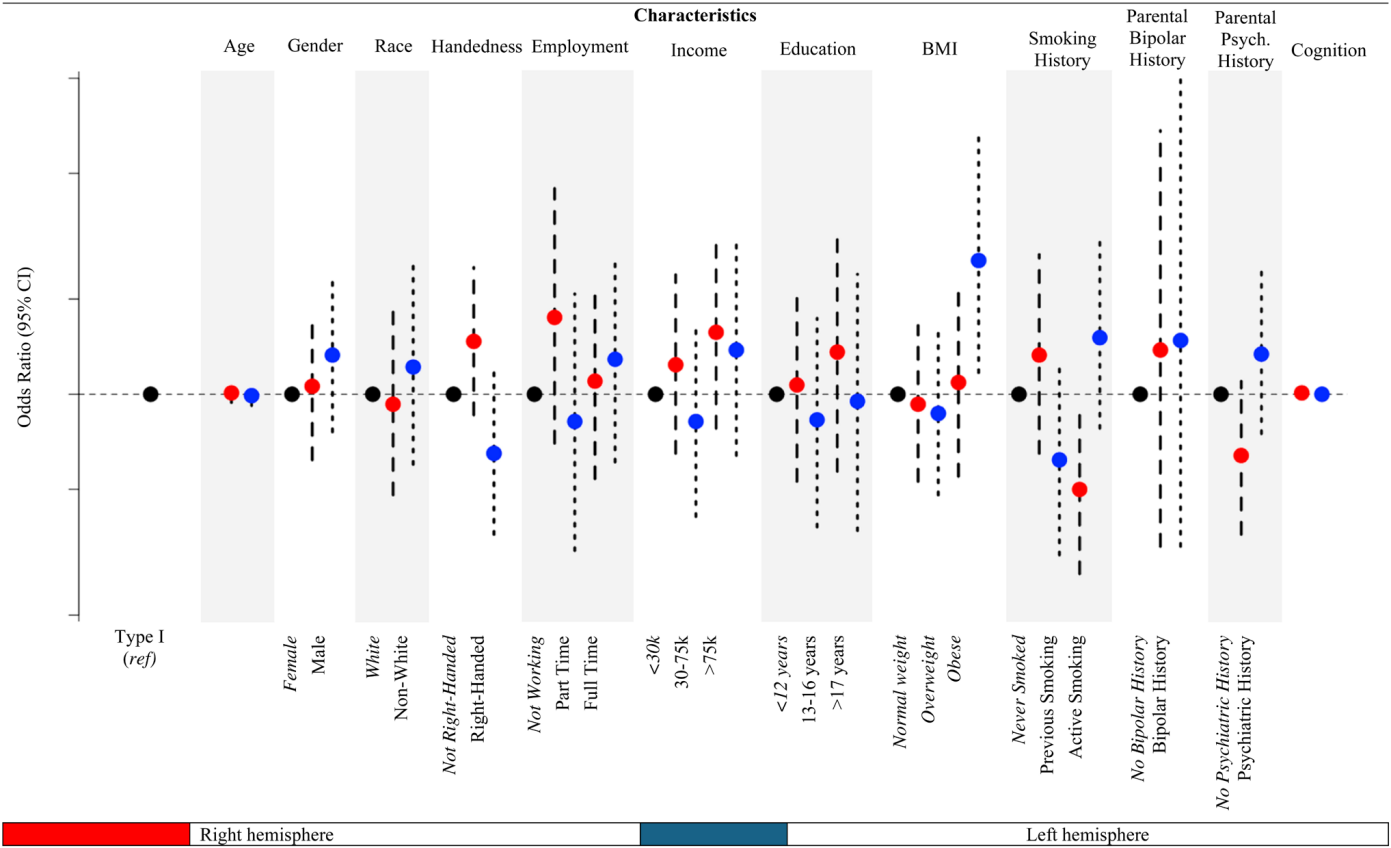
Bivariate associations of study sample characteristics with OFC pattern types. OFC: orbitofrontal sulcus, BMI: body mass index. Odds ratios and 95% confidence intervals for the associations between individual covariates and OFC pattern type. Type I OFC pattern type serves as the reference group (compared with other pattern types: including Type II, Type III, and Type IV). Type I pattern has a discontinuous MOS and continuous LOS, Type II a continuous MOS and continuous LOS, Type III a discontinuous MOS and discontinuous LOS, and Type IV a continuous MOS and discontinuous LOS. Note, y-axis is displayed on the log scale. Education categories are consistent with typical time for 1: less than high school or high school graduate, 2: some college or trade school, 3: advanced degree. For BMI categories, normal and underweight participants were grouped together, given the small sample size of those who are underweight (n = 6). Smoking history is based on self-report, where previous is defined as 1–99 times or experimental smoking. Parental psychiatric history is based on self-report of maternal and/or paternal psychiatric history. Any psychiatric history includes one or more of the following: schizophrenia or psychosis, depression, bipolar, anxiety, drug or alcohol problems, Alzheimer’s or dementia, Parkinson’s, Tourette’s syndrome. Cognition was evaluated using the Cognitive Function Composite Score National Institute of Health (NIH) toolbox, where higher scores indicate higher levels of cognitive functioning.

Overall, we did not observe that study sample characteristics were associated with odds of having an uncommon OFC pattern type (Type II, Type III, or Type IV) compared with Type I in either the right or left hemisphere, with few exceptions. For example, individuals who have obesity are 165% more likely to have an uncommon pattern type than Type I in the left hemisphere, compared with those who have normal or overweight BMIs (OR: 2.65; 95% CI:1.17, 6.65) (Fig. 4,Supplemental Table S4). Additionally, individuals who self-identified as regular smokers were 50% less likely or have a 0.50 decreased odds (OR: 0.50; 95% CI: 0.27, 0.92) of having an uncommon pattern type than Type I in the right hemisphere compared with those who self-identified as never smokers. Of note, observed associations were no longer significant following adjustment for multiple comparisons.

### 3.4 Secondary analyses

**Fig. 5.**
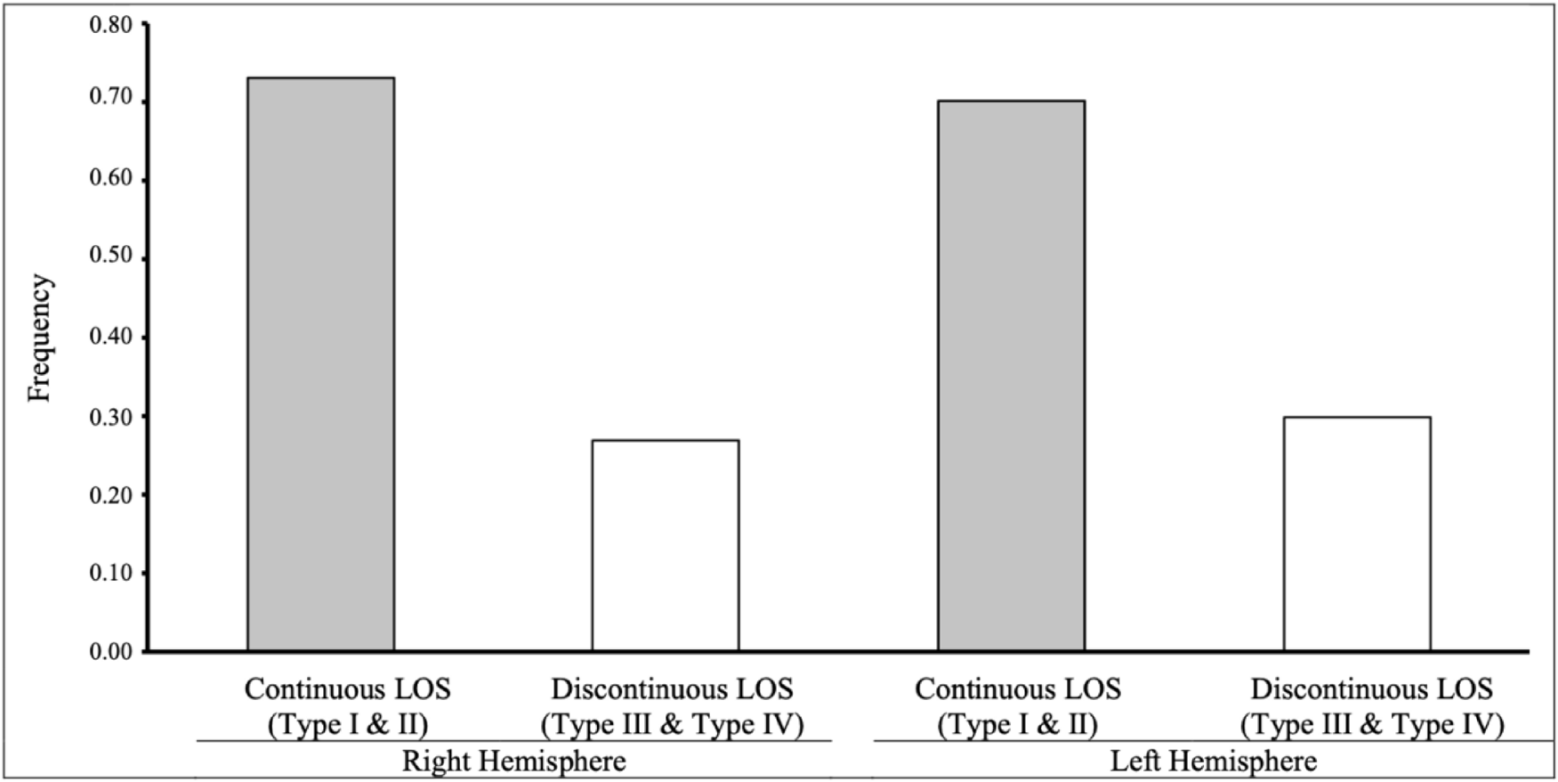
Joint hemispheric OFC pattern type frequency based on continuity and discontinuity of the LOS. OFC: orbitofrontal sulcus, LOS: lateral orbital sulcus. Patterns with a continuous LOS include Type I pattern (discontinuous MOS and continuous LOS) and Type II pattern (MOS and continuous LOS). Patterns with a discontinuous LOS include Type III pattern (discontinuous MOS and discontinuous LOS) and Type IV pattern (continuous MOS and discontinuous LOS). Note, our sample size includes n = 238 participants for a total of 476 hemispheres.

In both the right and left hemispheres, we observed that it was more common to have a continuous LOS, though this was not surprising based on prior observations that Type I and Type II pattern types occurred more frequently than Type III or Type IV (Fig. 5). Of note, we did not observe differences in the distribution of LOS continuity by hemisphere, in that both hemispheres were predominantly (~70%) consisting of pattern types with a continuous LOS.

When evaluating the distribution of study sample characteristics based on grouping OFC sulcal patterns by continuity of the LOS, we observe some differences from the original classification (ie., Type I v. other). For example, we observed differences in OFC pattern type classification by sex when comparing those with a continuous versus a discontinuous LOS, which were not apparent when comparing Type I with uncommon pattern types, or Type I to Type II, Type III, or Type IV (Supplemental Table S5). Results from logistic regression analyses showed substantially different patterns of results when grouping participants based on the continuity of the LOS compared with the primary results where Type I was compared with uncommon pattern types (Supplemental Table S6).

## 4 Discussion

Leveraging a large convenience sample of previously pattern typed nonpsychiatric participants from the HCP, we evaluated the patterns and predictors of OFC sulcogyral pattern type by participant demographics, socioeconomic factors, and health history-related factors. We did not observe substantial variation in the distribution of demographic or socioeconomic characteristics by OFC sulcogyral patterns. However, we did observe some evidence that health-related factors, namely BMI and self-reported smoking history, were associated with odds of OFC sulcogyral pattern type. We did not observe similar patterns of association when we categorized participants based on the continuity of the LOS, suggesting that pattern typing is only meaningful when considering the continuity of both the LOS and MOS. The findings from this work have implications for future study designs, such as determining covariates for adjustment in statistical analysis. Future work would benefit from exploring the demographic, socioeconomic, and health history-related patterns and predictors of OFC sulcogyral pattern types in psychiatric patient samples.

Little prior work has considered how demographic, socioeconomic, or health history-related factors are associated with OFC sulcogyral pattern types. While assumptions have been made, suggesting lower socioeconomic position and lower cognitive abilities are associated with less common pattern types, similar to some psychiatric diagnoses, this has not been consistently reported or evaluated within control samples (Nakamura et al., 2007;Patti et al., 2020). Disentangling the complex associations between psychiatric diagnoses, participant characteristics, and OFC sulcogyral pattern types will be essential in furthering our understanding of the role OFC structural morphology plays in psychopathology. For example, the observed association between less common pattern types and lower socioeconomic position could be indirectly attributed to underlying psychopathology, such that individuals with more profound psychiatric symptoms may be less likely to hold consistent employment (Eaton et al., 2008;Patti & Troiani, 2018). Of note, within our sample of control participants, we did not observe evidence that employment or income was significantly associated with odds of OFC pattern type. For some participant characteristics, such as race, we have no reason to believe that distributions of OFC pattern types will vary across categories of a socially constructed variable (Patti et al., 2020). Indeed, differences in psychiatric diagnoses by racial group can be widely attributed to systematic practices in the medical system, access to mental health care, and/or trauma resulting from racism, not underlying biology (Harnett, 2020). Understanding the relationship between participant characteristics and OFC pattern types can provide clarity in future study design and analysis, such as determining covariates to adjust for or match participants in case–control designs. For example, it is inappropriate to adjust for a covariate that is not associated with both the exposure (i.e., OFC pattern type) and outcome (i.e., psychiatric diagnosis), and such adjustment may even induce bias into effect estimates (Robins & Greenland, 1992).

In addition to our main goal of describing the patterns and predictors of OFC sulcogyral pattern types, we also evaluated distributions of study sample demographic, socioeconomic, and health history-related characteristics based only on the continuity of the LOS. While the four individual OFC pattern types have been previously established (Chakirova et al., 2010;Chiavaras & Petrides, 2000), in some studies, Types III and IV have been categorized together, based on the similar characteristics of a discontinuous LOS, rare prevalence, and associations with psychopathology (Patti et al., 2020). This suggests that the discontinuity of the LOS may be meaningful in its association with psychopathology, though the continuity of the LOS as an individual brain feature (combined Types I and II) has not been previously evaluated. Here, we observed that the overall pattern, direction, and magnitude of associations for the comparison of continuous (Types I and II) versus discontinuous (Type III and IV) LOS did not match those from our primary analysis (comparing “common” Type I with “less common” Types II, III, and IV). This may suggest that continuity of the LOS represents a fundamentally different subset of the population, and that there is meaning in considering both the LOS and MOS in determining OFC pattern classification and its association with psychopathology. Thus, we cannot interpret the individual findings from these secondary analyses, as they are subject to misclassification bias. Further, these results also were contrary to our initial hypotheses regarding the impact of some demographic characteristics on OFC pattern type. For example, we have no reason to believe that race or gender would impact OFC pattern type (Patti et al., 2020;Uehara-Aoyama et al., 2011). While we do see some results that support the hypothesis that uncommon pattern types are associated with increased risk of psychopathology (i.e., increased odds of discontinuous LOS among those with a parental history of bipolar disorder (left hemisphere) or any psychiatric history (right hemisphere)), we speculate this is primarily driven by the strong associations with Type III and Type IV with psychopathology, as found in prior work.

Distributions of demographic, socioeconomic, and most health history-related characteristics did not vary by OFC sulcogyral pattern, with the exception of BMI and smoking history. These characteristics are of particular interest given the role of the OFC in reward processing and atypical reward valuation (Hornak et al., 2004). We observed that in the left hemisphere only (null results in the right hemisphere), the odds of having Types II, III, or IV, compared with Type I, were significantly elevated among those with obesity relative to those with normal BMIs. Although it should be emphasized that BMI and obesity are influenced by multiple factors, including genetics, there is some component of obesity that is thought to be diet induced, with overconsumption and excess weight resulting from hedonic eating driven by the reward value of food rather than metabolic need (Ross et al., 2020;Yan et al., 2017). We speculate the association between having obesity and less common OFC pattern types could be attributed to unhealthy relationships with food and reward processing, which could manifest as overeating (Shott et al., 2015). Additionally, there is some evidence that obesity sometimes co-occurs with psychopathology. For example, higher BMI is associated with diagnoses of autism spectrum disorder (Dhaliwal et al., 2019) and bipolar disorder (Liu et al., 2022). We also observed that in the right hemisphere, participants who self-identified as regular smokers were less likely to have Types II, III, or IV, compared with Type I relative to self-reported never smokers. This was unexpected, as we would have anticipated a regular smoker to be more likely to have uncommon pattern types, given the role of the OFC in reward system processing, and how altered reward systems are implicated in substance dependencies (Bracht et al., 2021). However, for some psychiatric diagnoses, including Parkinson’s disease, there is some evidence that smoking may be protective (Allam et al., 2004;Noyce et al., 2012;Wirdefeldt et al., 2011).

While we did not observe any associations between OFC patterns and age, it is still unclear how the process of aging, specifically dementia, may impact the manifestation and presentation of OFC patterns over time. It is believed that sulcogyral morphology is established during gestation (Cachia et al., 2021;Chi et al., 1977;Dubois et al., 2008,^2019^;Habas et al., 2012;Rajagopalan et al., 2011) and remains stable over time (Armstrong et al., 1995). However, longitudinal assessments and reproducibility of OFC pattern type have never been formally evaluated. One study observed differences in age between those with schizophrenia and controls, where cases had an elevated frequency of less common OFC pattern types, but dismissed the potential impact of age in OFC pattern type classification (Lavoie et al., 2014). Given that OFC folding patterns are likely developed in utero, and how early brain development has lasting impacts on long-term behavior and psychopathology risk, we speculate that any observed age difference is the result of selection bias in case/control recruitment. Further, given the association between less common OFC pattern types and psychopathology broadly, it is possible that association could be observed with dementia, though any observed associations are likely driven by underlying brain dysfunction and less due to severe atrophy altering the presence of OFC pattern formation.

We did not observe significant associations between OFC sulcogyral pattern type and parental history of psychiatric diagnoses. This was somewhat unexpected given patterns of familial heritability of psychopathology (Giangrande et al., 2022). We speculate the lack of association between pattern type and parental history of psychiatric diagnoses could be attributed to the relatively small sample size of participants with parental history of psychiatric diagnoses, further evidenced by large and imprecise confidence intervals. Study participants with psychiatric diagnoses were excluded from the sample (by HCP study design), thus it is possible that those with a higher likelihood of parental history of psychopathology were also excluded, emphasizing the need to conduct this work within a psychiatric, case sample. Further, prior work has identified elevated frequencies of less common pattern types among those with some psychiatric diagnoses, such as schizophrenia, bipolar disorder, autism spectrum disorder, and gambling disorder (Li et al., 2019;Patti & Troiani, 2018;Watanabe et al., 2014), but not consistently across all psychiatric diagnoses (including obsessive compulsive disorder, cannabis use disorder, or cocaine use disorder) (Chye et al., 2017;Delahoy et al., 2019;Patti et al., 2020). The comprehensive, and perhaps imprecise, category of parental history of psychopathology included psychiatric diagnoses which have not been previously evaluated for their association with OFC pattern types (i.e., Tourette’s syndrome), and may have diluted the overall effect of parental psychiatric history and OFC pattern type.

There are several strengths and limitations that should be considered in the context of these findings. First, it is a strength of this work that we leveraged existing data from a large, diverse cohort study, the HCP. It is also a strength that all participants were previously OFC sulcogyral pattern typed using semiautomated methods (Troiani et al., 2022). This work also has some limitations. First, our sample consisted entirely of nonpsychiatric control participants, by design of the HCP. Future work may consider similar analyses within a psychiatric population or the potential for psychiatric diagnosis to modify the relationship between demographic characteristics and OFC sulcogyral patterns. Second, we were limited to the demographic, socioeconomic, and health history-related characteristics previously collected by the HCP. Future work should expand upon the characteristics evaluated here, particularly among health history factors that have been related to psychopathology. For example, given the evidence for increased frequency of atypical sulcogyral patterns in schizophrenia, future work may consider health-related factors commonly associated with schizophrenia, such as diabetes and gastrointestinal issues (Colijn, 2022;Mamakou et al., 2018). Third, the majority of covariates considered in our analyses, particularly those where we observed primary results, were ascertained via participant self-report. Indeed, participant self-reported height, weight, and smoking history are notoriously misreported, bringing up concerns for reporting or recall bias, and misclassification (Moore et al., 2019;Roystonn et al., 2021). Fourth, we artificially selected for a more diverse sample of OFC pattern types by selecting for uncommon pattern types (Types II, III, and IV) from among twin pairs. While this allowed us to evaluate distributions of participant characteristics within a more diverse sample of OFC pattern types, this is not a random, representative sample. Fifth, observed associations were modest, at best, and did not remain significant following adjustment for multiple comparison testing. However, given the exploratory and descriptive nature of this work, we believe there is value in observing overall patterns and direction of associations rather than limiting our interpretation to hypothesis testing, consistent with recommendations from the American Statistical Association (Amrhein et al., 2019;Wassersteina & Lazara, 2016). Sixth, recruitment procedures for the original Human Connectome Project were designed specifically to enroll twins through the Missouri Family Registry. Given the unique developmental trajectories experienced by twins and multiples during gestation (i.e., increased risk of preterm birth), these findings are not necessarily generalizable to the broader population (Elam et al., 2021). Finally, our analyses evaluated associations between participant characteristics and OFC sulcogyral patterns of a single hemisphere only. We were underpowered to explore how combinations of different OFC sulcogyral patterns may have altered our overall pattern of results. Future work may consider this in larger samples, with a more diverse distribution of uncommon pattern types.

## 5 Conclusion

The frequencies of some sulcogyral patterns in the OFC have been previously associated with psychopathology, and while claims have been made about their associations with some participant characteristics, this has not been formally evaluated. Leveraging a large, diverse, previously OFC pattern typed subset of the HCP dataset, we evaluated patterns and predictors of OFC sulcogyral patterns with participant demographics, socioeconomic factors, and health history-related factors using multinomial regression. We did not observe associations between demographic, socioeconomic, or most health history-related factors with OFC patterns, with the exception of BMI and smoking history. Future studies should expand upon this work in psychiatric samples and consider how psychopathology may modify the relationship between participant characteristics and OFC sulcogyral pattern.

## Supplementary Material

Supplementary Material

## Data Availability

Structural images were obtained from the Human Connectome Project (HCP), a public use dataset. More information can be found athttps://www.humanconnectome.org/. Code for all reported analyses will be made available by request to the corresponding author.
