# Supplementary material for: Patterns and predictors of orbitofrontal sulcogyral morphology in a nonclinical population
Source: Imaging Neurosci (Camb). 2024 Dec 19;2:imag-2-00389. doi: 10.1162/imag_a_00389 (PMC12315727; doi:10.1162/imag_a_00389)
Supplement: Supplementary Material [file imag_a_00389-supp.pdf]

Supplemental Materials

Patterns and Predictors of Orbitofrontal Sulcogyral Morphology in a Non-Clinical Population

TABLE OF CONTENTS	Page #
**Table S1**. Frequencies of orbitofrontal cortex pattern types…………………………..	2
**Table S2**. Description of participant characteristics by hemisphere pattern type, including all pattern types…………………………………………………………...	3
**Table S3**. Distribution of participant characteristics from the excluded sample of twin participants…………………………………………………………………………..	5
**Table S4**. Bivariate associations of study sample characteristics with OFC pattern types……………………………………………………………………………………….	7
**Table S5**. Description of participant characteristics by hemisphere pattern type, characterized by the continuity or discontinuity of the LOS…………………………...	8
**Table S6**. Bivariate associations of study sample characteristics with OFC pattern types, characterized by the continuity or discontinuity of the LOS……………………	10

Table S1. Frequencies of orbitofrontal cortex pattern types[1]

	Full Cohort[2]	Analytic Sample[3]	Excluded Sample[4]
	N (%)	N (%)	N (%)
Total	476 (100)	238 (100)	238 (100)
Right Hemisphere			
Type I	276 (58)	107 (45)	169 (71)
Type II	99 (21)	67 (28)	32 (13)
Type III	85 (18)	55 (23)	30 13)
Type IV	16 (3.4)	9 (3.8)	7 (2.9)
Left Hemisphere			
Type I	225 (47)	78 (33)	147 (62)
Type II	132 (28)	89 (37)	43 (18)
Type III	87 (18)	51 (21)	36 (15)
Type IV	32 (6.7)	20 (8.4)	12 (5.0)
Both Hemispheres			
Type I & Type I	140 (29)	22 (9.2)	118 (50)
Type I & Type II	116 (24)	75 (32)	41(17)
Type I & Type III	89 (19)	54 (23)	35 (15)
Type I & Type IV	16 (3.4)	12 (5.0)	4 (1.7)
Type II & Type II	36 (7.6)	27 (11)	9 (3.8)
Type II & Type III	27 (5.7)	17 (7.1)	10 (4.2)
Type II & Type IV	16 (3.4)	10 (4.2)	6 (2.5)
Type III & Type III	21 (4.4)	14 (5.9)	7 (2.9)
Type III & Type IV	14 (2.9)	7 (2.9)	7 (2.9)
Type IV & Type IV	1 (0.2)	0 (-)	1 (0.4)
Both Hemispheres			
Type I & Type I	140 (29)	22 (9.2)	118 (50)
Type I & Other[5]	221 (46)	141 (59)	80 (34)
Other & Other	115 (24)	75 (32)	40 (17)

[1] The Type I pattern has a discontinuous MOS and continuous LOS, the Type II a continuous MOS and continuous LOS, the Type III a discontinuous MOS and discontinuous LOS, and Type IV a continuous MOS and discontinuous LOS.
[2] Full cohort includes all twin pairs
[3] Includes only one participant from each twin pair, included in the analytic sample
[4] Includes only one participant from each twin pair, the excluded participant from the analytic sample
[5] Other pattern type refers to uncommon patterns: Type II, Type III, and Type IV

Table S2. Description of participant characteristics by hemisphere pattern type, including all pattern types

	Right Hemisphere			Left Hemisphere		
	Type I	Type II	Type III & Type IV	Type I	Type II	Type III & Type IV
Variable	N (%)	N (%)	N (%)	N (%)	N (%)	N (%)
	107	67	64	78	89	71
Age						
<25	9 (8.4)	9 (13)	12 (19)	7 (9.0)	10 (11)	23 (32)
25-30	46 (43)	35 (39)	28 (39)	32 (41)	38 (43)	40 (56)
30-35	45 (42)	19 (28)	37 (58)	35 (45)	36 (40)	42 (59)
>35	7 (6.5)	4 (6.0)	14 (22)	4 (5.1)	5 (5.6)	26 (37)
Gender						
Female	66 (62)	35 (52)	44 (69)	51 (65)	43(48)	51 (72)
Male	41 (38)	32 (48)	20 (31)	27 (35)	46 (52)	20 (28)
Race						
White	88 (82)	59 (88)	50 (78)	66 (85)	77 (87)	54 (76)
Black	13 (12)	5 (7.5)	8 (13)	6 (7.7)	10 (11)	10 (14)
Other [a]	6 (5.6)	3 (4.5)	6 (9.4)	6 (7.7)	2 (2.2)	7 (9.9)
Ethnicity						
Hispanic or Latino	3 (2.8)	3 (4.5)	0 (--)	3 (3.8)	2 (2.2)	1 (1.4)
Not Hispanic or Latino	103 (96)	63 (94)	63 (98)	74 (95)	87 (97.8)	68 (95.8)
Unknown or Not Reported	1 (0.9)	1 (1.5)	1 (1.6)	1 (1.3)	0 (--)	2 (2.8)
Employment						
Full time	75 (70)	41 (61)	46 (72)	49 (63)	64 (72)	49 (69)
Part time	13 (12)	17 (25)	7 (11)	15 (19)	14 (16)	8 (11)
Not working	19 (18)	9 (13)	11 (17)	14 (18)	11 (12)	14 (20)
Household Income						
<$30k	28 (26)	15 (22)	12 (19)	18 (23)	19 (21)	18 (25)
$30k-<$75k	29 (27)	19 (28)	25 (39)	19 (24)	34 (38)	20 (28)
>$75k	50 (47)	33 (49)	27 (42)	41 (53)	36 (40)	33 (46)
Education [b]						
<12 years	19 (18)	11 (16)	10 (16)	12 (15)	17 (19)	11 (15)
13-16 years	70 (65)	48 (72)	35 (55)	52 (67)	56 (63)	45 (63)
17+ years	18 (17)	8 (12)	19 (30)	14 (18)	16 (18)	15 (21)
Handedness						
Right-handed	65 (61)	45 (67)	46 (72)	56 (72)	52 (58)	48 (68)
Not right-handed	42 (39)	22 (33)	18 (28)	22 (28)	37 (42)	23 (32)
BMI						
Normal or underweight [c]	47 (44)	29 (43)	29 (45)	37 (47)	37 (42)	31 (44)
Overweight	40 (37)	25 (37)	21 (33)	33 (42)	32 (36)	21 (30)
Obese	20 (19)	13 (19)	14 (22)	8 (10)	20 (22)	19 (27)
Smoking History [d]						
Never smoked	54 (50)	38 (57)	38 (59)	43 (55)	49 (55)	38 (54)
Previous or some smoking	15 (14)	14 (21)	14 (22)	19 (24)	11 (12)	13 (18)
Regular smoking	38 (36)	15 (22)	12 (19)	16 (21)	29 (33)	20 (28)
Parental Psychiatric History [e]						
History of bipolar disorder [f]	3 (2.8)	2 (3.0)	3 (4.7)	2 (2.6)	1 (1.1)	3 (4.2)
No parental psychiatric history [g]	68 (64)	45 (67)	51 (80)	57 (73)	60 (67)	57 (80)
Any parental psychiatric history	39 (36)	22 (33)	13 (20)	21 (27)	29 (33)	24 (34)

[a] includes “more than one”, “unknown/unreported”, and “Asian/native Hawaiian/other pacific islander”
[b] Consistent with typical time for 1: less than high school or high school graduate”, 2: some college or trade school, 3: advanced degree
[c] only n=6 were underweight
[d] previous or smoke smoking (1-99 times, experimental smoking)

[e] Mother or father psychiatric history
[f] Mother history (n=5), father history (n=3)
[g] psychiatric history includes one of the following: schizophrenia or psychosis, depression, bipolar, anxiety, drug or alcohol problems, Alzheimer's or dementia, Parkinson's, Tourette's Syndrome
Note, The Type I pattern has a discontinuous MOS and continuous LOS, the Type II a continuous MOS and continuous LOS, the Type III a discontinuous MOS and discontinuous LOS, and Type IV a continuous MOS and discontinuous LOS.

Table S3. Description of participant characteristics from the excluded sample of twin participants

Variable	Full Sample N (%)
	238 (100)
Age	
<25	21 (8.9)
25-30	100 (42)
30-35	100 (42)
>35	17 (7.1)
Gender	
Female	145 (61)
Male	93 (39)
Race	
White	197 (83)
Black	26 (11)
Other [a]	15 (6.3)
Ethnicity	
Hispanic or Latino	6 (2.5)
Not Hispanic or Latino	229 (96)
Unknown or Not Reported	3 (1.3)
Employment	
Full time	176 (74)
Part time	33 (14)
Not working	29 (12)
Household Income	
<$30k	59 (25)
$30k-<$75k	84 (35)
>$75k	95 (40)
Education [b]	
<12 years	41 (17)
13-16 years	154 (65)
17+ years	43 (18)
Handedness	
Right-handed	153 (64)
Not right-handed	85 (36)
BMI	
Normal or underweight [c]	115 (48)
Overweight	72 (30)
Obese	51 (21)
Smoking History [d]	
Never smoked	135 (57)
Previous or some smoking	47 (20)
Regular smoking	56 (24)
Parental Psychiatric History [e]	
History of bipolar disorder [f]	8 (3.4)
No parental psychiatric history [g]	164 (69)
Any parental psychiatric history	74 (31)

OFC: orbitofrontal cortex; BMI: body mass index
[a] Includes "more than one", "unknown/unreported", and "Asian/native Hawaiian/other pacific islander"
[b] Consistent with typical time for: less than high school or high school graduate, some college or trade school, advanced degree
[c] Only n=6 participants were underweight

[d] Previous or smoke smoking (1-99 times, experimental smoking)
[e] Mother or father psychiatric history
[f] Mother history (n=5), father history (n=3)
[g] Psychiatric history includes one of the following: schizophrenia or psychosis, depression, bipolar, anxiety, drug or alcohol problems, Alzheimer’s or dementia, Parkinson’s, Tourette’s Syndrome

Table S4. Bivariate associations of study sample characteristics with OFC pattern types.

	Right Hemisphere				Left Hemisphere			
	Type I	Other			Type I	Other		
Variable	1.00 (reference) [b]	OR (95% CI) [a]	P-value	Adjusted P-value	1.00 (reference)	OR (95% CI)	P-value	Adjusted P-value
Age (continuous)	1.00	1.01 (0.94, 1.09)	0.77	0.99	1.00	0.99 (0.92, 1.08)	0.83	0.99
Gender				0.99				
Female		Ref				Ref		
Male	1.00	1.06 (0.62, 1.79)	0.83	0.99	1.00	1.33 (0.76, 2.35)	0.33	0.99
Race								
White		Ref				Ref		
Non-White	1.00	0.93 (0.48, 1.85)	0.85	0.99	1.00	1.22 (0.60, 2.62)	0.60	0.99
Handedness								
Not right-handed		Ref				Ref		
Right-handed	1.00	1.47 (0.86, 2.52)	0.16	0.99	1.00	0.65 (0.36, 1.17)	0.16	0.99
Employment								
Not working		Ref				Ref		
Part time	1.00	1.75 (0.70, 4.48)	0.23	0.99	1.00	0.82 (0.32, 2.08)	0.68	0.99
Full time	1.00	1.10 (0.54, 2.22)	0.79	0.99	1.00	1.29 (0.61, 2.67)	0.50	0.99
Household Income								
<$30k		Ref				Ref		
$30k-<$75k	1.00	1.24 (0.65, 2.39)	0.51	0.99	1.00	0.82 (0.41, 1.61)	0.57	0.99
>$75k	1.00	1.57 (0.78, 3.21)	0.21	0.99	1.00	1.38 (0.64, 2.99)	0.41	0.99
Education [c]								
<12 years		Ref				Ref		
13-16 years	1.00	1.07 (0.53, 2.16)	0.84	0.99	1.00	0.83 (0.38, 1.74)	0.63	0.99
17+ years	1.00	1.36 (0.57, 3.23)	0.49	0.99	1.00	0.95 (0.37, 2.40)	0.91	0.99
BMI								
Normal or underweight [d]		Ref				Ref		
Overweight	1.00	0.93 (0.53, 1.65)	0.81	0.99	1.00	0.87 (0.48, 1.58)	0.65	0.99
Obese	1.00	1.09 (0.55, 2.21)	0.80	0.99	1.00	2.65 (1.17, 6.65)	0.03	0.51
Smoking History [e]								
Never smoked		Ref				Ref		
Previous or some smoking	1.00	1.33 (0.65, 2.77)	0.44	0.99	1.00	0.62 (0.31, 1.27)	0.19	0.99
Regular smoking	1.00	0.50 (0.27, 0.92)	0.03	0.51	1.00	1.51 (0.78, 3.03)	0.23	0.99
Parental Psychiatric History [f]								
No history of bipolar		Ref				Ref		
History of bipolar disorder [g]	1.00	1.38 (0.33, 6.83)	0.67	0.99	1.00	1.48 (0.33, 10.27)	0.64	0.99
No parental psychiatric history		Ref				Ref		
Any parental psychiatric history [h]	1.00	0.64 (0.36, 1.10)	0.11	0.99	1.00	1.34 (0.75, 2.48)	0.33	0.99
Total Cognition T-score [i]	1.00	1.01 (0.99, 1.02)	0.34	0.99	1.00	1.00 (0.98, 1.01)	0.57	0.99

OFC: orbitofrontal sulcus, BMI: body mass index.

[a] Odds ratios and 95% confidence intervals for the associations between individual covariates and OFC pattern type.

[b] Type I OFC pattern type serves as the reference group (compared to other pattern type: including Type II, Type III, and Type IV). The Type I pattern has a discontinuous MOS and continuous LOS, the Type II a continuous MOS and continuous LOS, the Type III a discontinuous MOS and discontinuous LOS, and Type IV a continuous MOS and discontinuous LOS.

[c] Consistent with typical time for 1: less than high school or high school graduate", 2: some college or trade school, 3: advanced degree

[d] Only n=6 participants were underweight

[f] Previous or smoke smoking (1-99 times, experimental smoking)

[g] Mother or father psychiatric history

[h] Psychiatric history includes one of the following: schizophrenia or psychosis, depression, bipolar, anxiety, drug or alcohol problems, Alzheimer's or dementia, Parkinson's, Tourette's Syndrome

[I] Cognition was evaluated using the Cognitive Function Composite Score National Institute of Health (NIH) toolbox, where higher scores indicate higher levels of cognitive functioning.

Note, these results correspond to Figure 4.

Table S5. Description of participant characteristics by hemisphere pattern type, characterized by the continuity or discontinuity of the LOS

Variable	Right Hemisphere		Left Hemisphere	
	Continuous LOS	Discontinuous LOS	Continuous LOS	Discontinuous LOS
	N (%)	N (%)	N (%)	N (%)
	174	64	167	71
Age				
<25	18 (10)	3 (5)	17 (10)	4 (6)
25-30	81 (47)	19 (30)	70 (42)	30 (42)
30-35	64 (37)	36 (56)	71 (43)	29 (41)
>35	11 (6)	6 (9)	9 (5)	8 (11)
Gender				
Female	101 (58)	44 (69)	94 (56)	51 (72)
Male	73 (42)	20 (31)	73 (44)	20 (28)
Race				
White	147 (85)	50 (78)	143 (86)	54 (76)
Black	18 (10)	8 (13)	16 (10)	10 (14)
Other [a]	9 (5)	6 (9)	8 (5)	7 (10)
Ethnicity				
Hispanic or Latino	6 (3)	0 (-)	5 (3)	1 (1)
Not Hispanic or Latino	166 (95)	63 (98)	161 (96)	68 (96)
Unknown or Not Reported	2 (1)	1 (2)	2 (1)	2 (3)
Handedness				
Right-handed	110 (63)	46 (72)	108 (65)	59 (83)
Not right-handed	64 (37)	18 (28)	48 (29)	23 (32)
Employment				
Full time	116 (67)	46 (72)	113 (68)	49 (69)
Part time	30 (17)	7 (11)	29 (17)	8 (11)
Not working	28 (16)	11 (17)	25 (15)	14 (20)
Household Income				
<$30k	43 (25)	12 (19)	37 (22)	18 (25)
$30k-<$75k	48 (28)	25 (39)	53 (32)	20 (28)
>$75k	83 (48)	27 (42)	77 (46)	33 (46)
Education [b]				
<12 years	30 (17)	10 (16)	29 (17)	11 (15)
13-16 years	118 (68)	35 (55)	108 (65)	45 (63)
17+ years	26 (15)	19 (30)	30 (18)	15 (21)
BMI				
Normal or underweight [c]	76 (44)	29 (45)	74 (44)	31 (44)
Overweight	65 (37)	21 (33)	65 (39)	21 (30)
Obese	33 (19)	14 (22)	28 (17)	19 (27)
Smoking History [d]				
Never smoked	92 (53)	38 (59)	92 (55)	38 (54)
Previous or some smoking	29 (17)	14 (22)	30 (18)	13 (18)
Regular smoking	53 (30)	12 (19)	45(27)	20 (28)
Parental Psychiatric History [e]				
History of bipolar disorder [f]	5 (3)	3 (5)	3 (2)	5 (7)
No psychiatric history	113 (65)	61 (95)	117 (70)	50 (70)
Any psychiatric history [g]	51 (29)	13 (20)	47 (28)	24 (34)

LOS: lateral orbital sulcus, BMI: body mas index
[a] includes “more than one”, “unknown/unreported”, and “Asian/native Hawaiian/other pacific islander”

[b] Consistent with typical time for 1: less than high school or high school graduate", 2: some college or trade school, 3: advanced degree
[c] only n=6 participants were underweight
[d] previous or smoke smoking (1-99 times, experimental smoking)
[e] Mother or father psychiatric history
[f] Mother history (n=5), father history (n=3)
[g] psychiatric history includes one of the following: schizophrenia or psychosis, depression, bipolar, anxiety, drug or alcohol problems, Alzheimer's or dementia, Parkinson's, Tourette's Syndrome
Note, Patterns with a continuous LOS include Type I pattern (discontinuous MOS and continuous LOS) and the Type II pattern (MOS and continuous LOS). Patters with a discontinuous LOS include the Type III pattern (discontinuous MOS and discontinuous LOS) and Type IV pattern (continuous MOS and discontinuous LOS).

Table S6. Bivariate associations of study sample characteristics with OFC pattern types, characterized by the continuity or discontinuity of the LOS

	Right Hemisphere			Left Hemisphere		
	Continuous LOS	Discontinuous LOS		Continuous LOS	Discontinuous LOS	
Variable	(ref) [b]	OR (95% CI) [a]	P-value	(ref)	OR (95% CI)	P-value
Age (continuous)	1.00	1.11 (1.02, 1.21)	0.02	1.00	1.03 (0.95, 1.12)	0.48
Gender						
Female		Ref			Ref	
Male	1.00	0.63 (0.34, 1.14)	0.14	1.00	0.50 (0.27, 0.91)	0.03
Race						
White		Ref			Ref	
Non-White	1.00	1.52 (0.73, 3.10)	0.25	1.00	1.88 (0.92, 3.75)	0.08
Handedness						
Not right-handed		Ref			Ref	
Right-handed	1.00	1.49 (0.81, 2.83)	0.21	1.00	1.14 (0.64, 2.08)	0.66
Employment						
Not working		Ref			Ref	
Part time	1.00	0.59 (0.19, 1.72)	0.34	1.00	0.49 (0.17, 1.34)	0.17
Full time	1.00	1.01 (0.47, 2.27)	0.98	1.00	0.77 (0.37, 1.65)	0.50
Household Income						
<$30k		Ref			Ref	
$30k-<$75k	1.00	1.17 (0.55, 2.56)	0.70	1.00	0.88 (0.44, 1.79)	0.72
>$75k	1.00	1.87 (0.85, 4.27)	0.13	1.00	0.78 (0.36, 1.67)	0.51
Education [c]						
<12 years		Ref			Ref	
13-16 years	1.00	0.89 (0.41, 2.08)	0.78	1.00	1.10 (0.52, 2.47)	0.81
17+ years	1.00	2.19 (0.88, 5.71)	0.10	1.00	1.32 (0.52, 3.40)	0.56
BMI						
Normal or underweight [d]		Ref			Ref	
Overweight	1.00	0.85 (0.44, 1.62)	0.62	1.00	0.77 (0.40, 1.47)	0.43
Obese	1.00	1.11 (0.51, 2.35)	0.78	1.00	1.62 (0.79, 3.32)	0.19
Smoking History [e]						
Never smoked		Ref			Ref	
Previous or some smoking	1.00	1.17 (0.55, 2.43)	0.68	1.00	1.05 (0.48, 2.20)	0.90
Regular smoking	1.00	0.55 (0.26, 1.11)	0.11	1.00	1.08 (0.56, 2.05)	0.83
Parental Psychiatric History [f]						
No history of bipolar		Ref			Ref	
History of bipolar disorder [g]	1.00	1.66 (0.33, 6.98)	0.50	1.00	4.14 (0.99, 20.66)	0.06
No psychiatric history		Ref			Ref	
Any psychiatric history [h]	1.00	0.47 (0.23, 0.91)	0.03	1.00	1.19 (0.65, 2.15)	0.56
Total Cognition T-score [i]	1.00	1.01 (0.99, 1.02)	0.33	1.00	0.99 (0.98, 1.00)	0.05

LOS: lateral orbital sulcus, BMI: body mass index.

[a] Odds ratios and 95% confidence intervals for the associations between individual covariates and OFC pattern type.

[b] Continuous LOS served as the reference group. Patterns with a continuous LOS include Type I pattern (discontinuous MOS and continuous LOS) and the Type II pattern (MOS and continuous LOS). Patters with a discontinuous LOS include the Type III pattern (discontinuous MOS and discontinuous LOS) and Type IV pattern (continuous MOS and discontinuous LOS).

[c] Consistent with typical time for 1: less than high school or high school graduate", 2: some college or trade school, 3: advanced degree

[d] Only n=6 participants were underweight

[e] Previous or smoke smoking (1-99 times, experimental smoking)

[g] Mother or father psychiatric history

[h] Psychiatric history includes one of the following: schizophrenia or psychosis, depression, bipolar, anxiety, drug or alcohol problems, Alzheimer's or dementia, Parkinson's, Tourette's Syndrome

[i] Cognition was evaluated using the Cognitive Function Composite Score National Institute of Health (NIH) toolbox, where higher scores indicate higher levels of cognitive functioning.
